# {3-[Bis(2-pyridyl­methyl-κ*N*)amino-κ*N*]propanol}bis­(nitrato-κ*O*)copper(II)

**DOI:** 10.1107/S1600536810051779

**Published:** 2010-12-15

**Authors:** Sankara Rao Rowthu, Jong Won Shin, Yu Rim Seo, Kil Sik Min

**Affiliations:** a Department of Chemistry, Kyungpook National University, Daegu 702-701, Republic of Korea; b Department of Chemistry Education, Kyungpook National University, Daegu 702-701, Republic of Korea

## Abstract

In the title compound, [Cu(NO_3_)_2_(C_15_H_19_N_3_O)], the Cu^II^ ion is coordinated by the N atoms of the tetra­dentate 3-[bis­(2-pyridyl­meth­yl)amino]­propanol ligand and two O atoms from two monodentate nitrate anions, resulting in a distorted square-pyramidal environment. An inter­molecular O—H⋯O hydrogen-bonding inter­action between the free hy­droxy group of the ligand and a nitrate O atom of an adjacent complex unit, gives a chain structure which extends across the (101) planes.

## Related literature

Polyamine complexes have been characterized in order to elucidate the mechanisms of metalloenzymes, see: Tshuva & Lippard (2004 ▶). For complexes with bis­(2-pyridyl­meth­yl)amine ligands, see: Bebout *et al.* (1998 ▶); Shin *et al.* (2010 ▶). Compounds with tridentate units have potential biological applications, see: van Staveren *et al.* (2002 ▶). Palladium(II) and platinum(II) complexes with bis­(2-pyridyl­meth­yl)amine or its derivatives have been investigated as potential anti­cancer agents including *cis*-platin (Rauterkus *et al.*, 2003 ▶). For the preparation of *N*,*N*-bis­(2-pyridyl­meth­yl)-3-amino­propanol, see: Young *et al.* (1995 ▶).
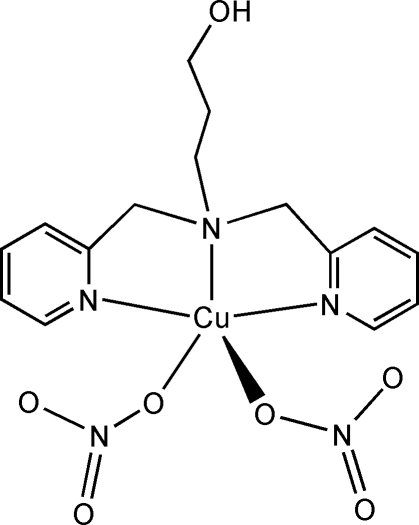


## Experimental

### 

#### Crystal data

[Cu(NO_3_)_2_(C_15_H_19_N_3_O)]
*M* *_r_* = 444.89
Monoclinic, 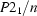

*a* = 8.3499 (7) Å
*b* = 14.7703 (12) Å
*c* = 14.5134 (12) Å
β = 95.055 (2)°
*V* = 1783.0 (3) Å^3^
*Z* = 4
Mo *K*α radiation
μ = 1.28 mm^−1^
*T* = 200 K
0.26 × 0.13 × 0.09 mm

#### Data collection

Siemens SMART CCD diffractometer
Absorption correction: multi-scan (*SADABS*; Sheldrick, 1996 ▶) *T*_min_ = 0.820, *T*_max_ = 0.892
13134 measured reflections
4412 independent reflections
2297 reflections with *I* > 2σ(*I*)
*R*_int_ = 0.078

#### Refinement

*R*[*F* ^2^ > 2σ(*F* ^2^)] = 0.053
*wR*(*F* ^2^) = 0.151
*S* = 1.04
4412 reflections
254 parameters
H-atom parameters constrained
Δρ_max_ = 0.78 e Å^−3^
Δρ_min_ = −0.66 e Å^−3^

### 

Data collection: *SMART* (Siemens, 1996 ▶); cell refinement: *SAINT* (Siemens, 1996 ▶); data reduction: *SAINT*; program(s) used to solve structure: *SHELXS97* (Sheldrick, 2008 ▶); program(s) used to refine structure: *SHELXL97* (Sheldrick, 2008 ▶); molecular graphics: *ORTEP-3* (Farrugia, 1997 ▶); software used to prepare material for publication: *SHELXL97*.

## Supplementary Material

Crystal structure: contains datablocks global, I. DOI: 10.1107/S1600536810051779/zs2083sup1.cif

Structure factors: contains datablocks I. DOI: 10.1107/S1600536810051779/zs2083Isup2.hkl

Additional supplementary materials:  crystallographic information; 3D view; checkCIF report

## Figures and Tables

**Table 1:** Hydrogen-bond geometry (Å, °)

*D*—H⋯*A*	*D*—H	H⋯*A*	*D*⋯*A*	*D*—H⋯*A*
O1—H1⋯O7^i^	0.84	2.18	2.961 (6)	155

Symmetry code: (i) 
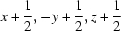
.

**Fig. 1.**
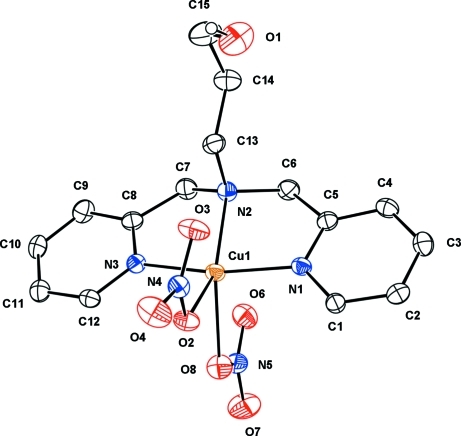
ORTEP drawing of the title compound with atomic numbering scheme and 30% probability ellipsoids.

**Fig. 2.**
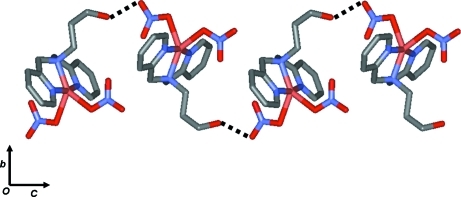
A view of the title compound showing a one-dimensional chain structure formed by O—H···O hydrogen-bonding interactions.

## supplementary crystallographic information

### Comment

The preparation and characterization of a large number of polyamine complexes has been done, in order to elucidate the mechanisms of metalloenzymes (Tshuva & Lippard, 2004). Recently, the complexes with bis(2-pyridylmethyl)amine moieties have been widely described (Bebout *et al.*,1998; Shin *et al.*, 2010) because the tridentate unit is a good candidate for potential biological applications (van Staveren *et al.*, 2002). For example, palladium(II) and platinum(II) complexes with bis(2-pyridylmethyl)amine or its derivatives have been investigated as potential anticancer agents, e.g. *cis*-platin (Rauterkus *et al.*, 2003). Here, we report the synthesis and crystal structure of five-coordinate Cu(NO_3_)_2_ complex with the tetradentate ligand *N*,*N*-bis(2-pyridylmethyl)-3-aminopropanol = bdap), the title compound [Cu(bpap)(NO_3_)_2_] (I), and the structure is reported here.

In the title compound (Fig. 1), the Cu^II^ ion is five-coordinated and exhibits a distorted square pyramidal geometry, the equatorial plane being defined by the three nitrogen atoms of the bdap ligand and one oxygen atom of a nitrate ion. The coordination geometry is completed by the axial coordination of the oxygen atom of the second nitrate anion. The Cu—*L*_eq_ bond lengths are in the range of 1.965 (4) and 2.093 (3) Å and the Cu—O_ax_ bond length is 2.248 (3) Å. Both nitrate ions are bound in *η*^1^-fashion. The bond angles about the copper atom range from 76.95 (12) to 165.48 (15)°. The packing structure involves a strong O—H···O hydrogen bonding interaction between the free hydroxyl group of the bpap ligand and a nitrate O atom of an adjacent complex unit (Table 1), giving a one-dimensional chain structure which extends across the (101) planes in the unit cell (Fig. 2).

### Experimental

A MeOH solution (5 ml) of Cu(NO_3_)_2_ . 3H_2_O (47 mg, 0.194 mmol) was added to a MeOH solution (5 ml) of *N*,*N*-bis(2-pyridylmethyl)-3-aminopropanol (bpap, 50 mg, 0.194 mmol) (Young *et al.*, 1995). The color changed to blue-green, and the solution was stirred for 10 min at room temperature. Blue-green crystals were obtained by diffusion of diethyl ether into the reaction mixture in methanol and were collected by filtration, washed with diethyl ether, and dried in air (yield: 36 mg, 42%). FTIR (KBr, cm^-1^): 3399, 1437, 3069, 2970, 2862, 1054, 1608.

### Refinement

All H atoms in the title compound were placed in geometrically idealized positions and constrained to ride on their parent atoms, with C—H distances of 0.95 (ring H atoms) or 0.99 (open chain H atoms) Å and an O—H distance of 0.84 Å, and with *U*_iso_(H) values of 1.2 or 1.5 times *U*_eq_(C,O).

### Crystal data

**Table:**

[Cu(NO_3_)_2_(C_15_H_19_N_3_O)]	*F*(000) = 916
*M**_r_* = 444.89	*D*_x_ = 1.657 Mg m^−^^3^
Monoclinic, *P*2_1_/*n*	Mo *K*α radiation, λ = 0.71073 Å
Hall symbol: -P 2yn	Cell parameters from 2504 reflections
*a* = 8.3499 (7) Å	θ = 2.7–23.6°
*b* = 14.7703 (12) Å	µ = 1.28 mm^−^^1^
*c* = 14.5134 (12) Å	*T* = 200 K
β = 95.055 (2)°	Needle, blue-green
*V* = 1783.0 (3) Å^3^	0.26 × 0.13 × 0.09 mm
*Z* = 4	

### Data collection

**Table:**

Siemens SMART CCD diffractometer	4412 independent reflections
Radiation source: fine-focus sealed tube	2297 reflections with *I* > 2σ(*I*)
graphite	*R*_int_ = 0.078
φ and ω scans	θ_max_ = 28.3°, θ_min_ = 2.0°
Absorption correction: multi-scan (*SADABS*; Sheldrick, 1996)	*h* = −11→11
*T*_min_ = 0.820, *T*_max_ = 0.892	*k* = −19→18
13134 measured reflections	*l* = −17→19

### Refinement

**Table:**

Refinement on *F*^2^	Primary atom site location: structure-invariant direct methods
Least-squares matrix: full	Secondary atom site location: difference Fourier map
*R*[*F*^2^ > 2σ(*F*^2^)] = 0.053	Hydrogen site location: inferred from neighbouring sites
*wR*(*F*^2^) = 0.151	H-atom parameters constrained
*S* = 1.04	*w* = 1/[σ^2^(*F*_o_^2^) + (0.0564*P*)^2^] where *P* = (*F*_o_^2^ + 2*F*_c_^2^)/3
4412 reflections	(Δ/σ)_max_ < 0.001
254 parameters	Δρ_max_ = 0.78 e Å^−^^3^
0 restraints	Δρ_min_ = −0.66 e Å^−^^3^

### Special details

**Table:**

Geometry. All e.s.d.'s (except the e.s.d. in the dihedral angle between two l.s. planes)are estimated using the full covariance matrix. The cell e.s.d.'s are takeninto account individually in the estimation of e.s.d.'s in distances, anglesand torsion angles; correlations between e.s.d.'s in cell parameters are onlyused when they are defined by crystal symmetry. An approximate (isotropic)treatment of cell e.s.d.'s is used for estimating e.s.d.'s involving l.s.planes.
Refinement. Refinement of *F*^2^ against ALL reflections. The weighted *R*-factor*wR* and goodness of fit *S* are based on *F*^2^, conventional*R*-factors *R* are based on *F*, with *F* set to zero fornegative *F*^2^. The threshold expression of *F*^2^ >σ(*F*^2^) is used only for calculating *R*-factors(gt) *etc*.and is not relevant to the choice of reflections for refinement.*R*-factors based on *F*^2^ are statistically about twice as largeas those based on *F*, and *R*- factors based on ALL data will beeven larger.

### Fractional atomic coordinates and isotropic or equivalent isotropic displacement parameters (Å^2^)

**Table:**

	*x*	*y*	*z*	*U*_iso_*/*U*_eq_	
Cu1	0.13958 (6)	0.34362 (4)	0.87320 (4)	0.03228 (19)	
N1	−0.0717 (4)	0.3222 (2)	0.9189 (2)	0.0314 (9)	
N2	0.1137 (4)	0.2127 (2)	0.8271 (2)	0.0336 (9)	
N3	0.3346 (4)	0.3396 (2)	0.8064 (2)	0.0320 (8)	
N4	0.2941 (4)	0.3812 (3)	1.0535 (2)	0.0344 (9)	
N5	0.0028 (5)	0.4753 (3)	0.7451 (3)	0.0464 (11)	
O1	0.2165 (5)	0.0185 (3)	1.0483 (3)	0.0818 (13)	
H1	0.2959	0.0168	1.0880	0.123*	
O2	0.2405 (4)	0.4250 (2)	0.9813 (2)	0.0396 (8)	
O3	0.2729 (4)	0.2988 (2)	1.0556 (2)	0.0539 (10)	
O4	0.3673 (4)	0.4229 (3)	1.1171 (2)	0.0614 (11)	
O5	0.0804 (4)	0.4846 (2)	0.8229 (2)	0.0497 (9)	
O6	−0.0202 (4)	0.3980 (3)	0.7120 (2)	0.0598 (10)	
O7	−0.0504 (5)	0.5423 (3)	0.7013 (3)	0.0789 (13)	
C1	−0.1437 (5)	0.3745 (3)	0.9779 (3)	0.0323 (10)	
H1A	−0.0927	0.4290	0.9992	0.039*	
C2	−0.2890 (5)	0.3519 (3)	1.0086 (3)	0.0373 (11)	
H2	−0.3394	0.3908	1.0496	0.045*	
C3	−0.3605 (6)	0.2721 (3)	0.9791 (3)	0.0401 (12)	
H3	−0.4592	0.2541	1.0015	0.048*	
C4	−0.2894 (5)	0.2181 (3)	0.9172 (3)	0.0382 (11)	
H4	−0.3384	0.1630	0.8957	0.046*	
C5	−0.1448 (5)	0.2458 (3)	0.8868 (3)	0.0335 (10)	
C6	−0.0626 (6)	0.1966 (3)	0.8132 (3)	0.0410 (12)	
H6A	−0.0849	0.1309	0.8165	0.049*	
H6B	−0.1046	0.2186	0.7513	0.049*	
C7	0.1975 (6)	0.2068 (3)	0.7424 (3)	0.0427 (12)	
H7A	0.1229	0.2238	0.6884	0.051*	
H7B	0.2328	0.1436	0.7336	0.051*	
C8	0.3421 (5)	0.2690 (3)	0.7485 (3)	0.0361 (11)	
C9	0.4694 (6)	0.2583 (3)	0.6956 (3)	0.0451 (13)	
H9	0.4763	0.2069	0.6569	0.054*	
C10	0.5875 (6)	0.3244 (3)	0.7002 (3)	0.0427 (12)	
H10	0.6751	0.3195	0.6629	0.051*	
C11	0.5779 (6)	0.3969 (3)	0.7584 (3)	0.0404 (11)	
H11	0.6579	0.4427	0.7616	0.049*	
C12	0.4513 (5)	0.4020 (3)	0.8115 (3)	0.0341 (10)	
H12	0.4458	0.4512	0.8532	0.041*	
C13	0.1911 (6)	0.1521 (3)	0.9023 (3)	0.0402 (11)	
H13A	0.1480	0.1690	0.9613	0.048*	
H13B	0.3079	0.1650	0.9089	0.048*	
C14	0.1684 (6)	0.0503 (3)	0.8885 (4)	0.0543 (14)	
H14A	0.0536	0.0348	0.8910	0.065*	
H14B	0.2000	0.0331	0.8267	0.065*	
C15	0.2670 (8)	−0.0015 (4)	0.9608 (4)	0.0659 (17)	
H15A	0.2553	−0.0672	0.9485	0.079*	
H15B	0.3818	0.0147	0.9594	0.079*	

### Atomic displacement parameters (Å^2^)

**Table:**

	*U*^11^	*U*^22^	*U*^33^	*U*^12^	*U*^13^	*U*^23^
Cu1	0.0363 (3)	0.0263 (3)	0.0347 (3)	−0.0016 (2)	0.0056 (2)	−0.0041 (2)
N1	0.034 (2)	0.026 (2)	0.034 (2)	−0.0022 (16)	0.0002 (17)	−0.0023 (15)
N2	0.035 (2)	0.033 (2)	0.032 (2)	−0.0039 (17)	0.0023 (17)	−0.0098 (16)
N3	0.033 (2)	0.033 (2)	0.030 (2)	0.0007 (17)	0.0041 (16)	0.0008 (16)
N4	0.037 (2)	0.037 (2)	0.030 (2)	−0.0022 (18)	0.0066 (18)	−0.0032 (18)
N5	0.047 (3)	0.039 (3)	0.054 (3)	0.004 (2)	0.007 (2)	0.010 (2)
O1	0.091 (3)	0.092 (4)	0.063 (3)	0.022 (3)	0.010 (2)	0.003 (2)
O2	0.054 (2)	0.0318 (19)	0.0322 (18)	−0.0032 (15)	0.0001 (15)	−0.0030 (14)
O3	0.073 (3)	0.030 (2)	0.056 (2)	−0.0080 (18)	−0.0066 (19)	0.0031 (17)
O4	0.082 (3)	0.062 (3)	0.037 (2)	−0.020 (2)	−0.0132 (19)	−0.0105 (17)
O5	0.055 (2)	0.053 (2)	0.040 (2)	0.0027 (17)	−0.0047 (18)	0.0028 (16)
O6	0.061 (2)	0.058 (3)	0.059 (2)	−0.009 (2)	0.0001 (19)	−0.001 (2)
O7	0.099 (3)	0.065 (3)	0.070 (3)	0.025 (2)	−0.006 (2)	0.034 (2)
C1	0.033 (3)	0.032 (3)	0.032 (2)	−0.0024 (19)	0.007 (2)	−0.0065 (19)
C2	0.041 (3)	0.036 (3)	0.035 (3)	0.006 (2)	0.002 (2)	−0.003 (2)
C3	0.037 (3)	0.048 (3)	0.036 (3)	−0.003 (2)	0.005 (2)	0.004 (2)
C4	0.042 (3)	0.031 (3)	0.040 (3)	−0.006 (2)	−0.003 (2)	−0.001 (2)
C5	0.035 (3)	0.033 (3)	0.032 (2)	0.003 (2)	0.000 (2)	0.0013 (19)
C6	0.041 (3)	0.036 (3)	0.045 (3)	−0.002 (2)	−0.004 (2)	−0.010 (2)
C7	0.046 (3)	0.039 (3)	0.043 (3)	−0.005 (2)	0.005 (2)	−0.012 (2)
C8	0.042 (3)	0.033 (3)	0.033 (3)	0.003 (2)	0.003 (2)	−0.004 (2)
C9	0.046 (3)	0.052 (3)	0.037 (3)	0.005 (3)	0.007 (2)	−0.012 (2)
C10	0.032 (3)	0.055 (4)	0.042 (3)	0.004 (2)	0.008 (2)	0.001 (2)
C11	0.037 (3)	0.046 (3)	0.038 (3)	0.001 (2)	0.003 (2)	0.006 (2)
C12	0.039 (3)	0.028 (3)	0.034 (3)	−0.002 (2)	−0.001 (2)	0.0002 (19)
C13	0.040 (3)	0.034 (3)	0.046 (3)	0.004 (2)	−0.002 (2)	−0.004 (2)
C14	0.066 (4)	0.038 (3)	0.059 (3)	0.001 (3)	0.004 (3)	−0.002 (2)
C15	0.091 (5)	0.055 (4)	0.053 (4)	0.021 (3)	0.015 (3)	0.008 (3)

### Geometric parameters (Å, °)

**Table:**

Cu1—N1	1.965 (4)	C3—H3	0.9500
Cu1—N3	1.968 (3)	C4—C5	1.383 (6)
Cu1—N2	2.051 (3)	C4—H4	0.9500
Cu1—O2	2.093 (3)	C5—C6	1.507 (6)
Cu1—O5	2.248 (3)	C6—H6A	0.9900
N1—C1	1.335 (5)	C6—H6B	0.9900
N1—C5	1.345 (5)	C7—C8	1.514 (6)
N2—C7	1.469 (5)	C7—H7A	0.9900
N2—C6	1.487 (5)	C7—H7B	0.9900
N2—C13	1.512 (6)	C8—C9	1.374 (6)
N3—C12	1.339 (5)	C9—C10	1.385 (6)
N3—C8	1.343 (5)	C9—H9	0.9500
N4—O4	1.227 (4)	C10—C11	1.370 (6)
N4—O3	1.230 (4)	C10—H10	0.9500
N4—O2	1.279 (4)	C11—C12	1.364 (6)
N5—O7	1.236 (5)	C11—H11	0.9500
N5—O6	1.247 (5)	C12—H12	0.9500
N5—O5	1.259 (5)	C13—C14	1.527 (6)
O1—C15	1.403 (6)	C13—H13A	0.9900
O1—H1	0.8400	C13—H13B	0.9900
C1—C2	1.370 (6)	C14—C15	1.487 (7)
C1—H1A	0.9500	C14—H14A	0.9900
C2—C3	1.373 (6)	C14—H14B	0.9900
C2—H2	0.9500	C15—H15A	0.9900
C3—C4	1.375 (6)	C15—H15B	0.9900
			
N1—Cu1—N3	165.48 (15)	N2—C6—C5	109.5 (4)
N1—Cu1—N2	83.45 (14)	N2—C6—H6A	109.8
N3—Cu1—N2	83.03 (14)	C5—C6—H6A	109.8
N1—Cu1—O2	98.85 (13)	N2—C6—H6B	109.8
N3—Cu1—O2	95.20 (13)	C5—C6—H6B	109.8
N2—Cu1—O2	144.03 (14)	H6A—C6—H6B	108.2
N1—Cu1—O5	94.64 (13)	N2—C7—C8	110.6 (4)
N3—Cu1—O5	92.06 (14)	N2—C7—H7A	109.5
N2—Cu1—O5	138.91 (14)	C8—C7—H7A	109.5
O2—Cu1—O5	76.95 (12)	N2—C7—H7B	109.5
C1—N1—C5	119.4 (4)	C8—C7—H7B	109.5
C1—N1—Cu1	126.3 (3)	H7A—C7—H7B	108.1
C5—N1—Cu1	114.3 (3)	N3—C8—C9	121.3 (4)
C7—N2—C6	114.5 (4)	N3—C8—C7	115.2 (4)
C7—N2—C13	111.3 (4)	C9—C8—C7	123.4 (4)
C6—N2—C13	111.0 (3)	C8—C9—C10	118.4 (4)
C7—N2—Cu1	106.5 (3)	C8—C9—H9	120.8
C6—N2—Cu1	105.6 (3)	C10—C9—H9	120.8
C13—N2—Cu1	107.2 (3)	C11—C10—C9	120.0 (4)
C12—N3—C8	119.6 (4)	C11—C10—H10	120.0
C12—N3—Cu1	125.6 (3)	C9—C10—H10	120.0
C8—N3—Cu1	114.7 (3)	C12—C11—C10	118.8 (5)
O4—N4—O3	122.8 (4)	C12—C11—H11	120.6
O4—N4—O2	118.5 (4)	C10—C11—H11	120.6
O3—N4—O2	118.6 (4)	N3—C12—C11	121.8 (4)
O7—N5—O6	119.9 (5)	N3—C12—H12	119.1
O7—N5—O5	120.4 (5)	C11—C12—H12	119.1
O6—N5—O5	119.7 (4)	N2—C13—C14	116.6 (4)
C15—O1—H1	109.5	N2—C13—H13A	108.1
N4—O2—Cu1	114.4 (3)	C14—C13—H13A	108.1
N5—O5—Cu1	105.7 (3)	N2—C13—H13B	108.1
N1—C1—C2	122.0 (4)	C14—C13—H13B	108.1
N1—C1—H1A	119.0	H13A—C13—H13B	107.3
C2—C1—H1A	119.0	C15—C14—C13	111.1 (4)
C1—C2—C3	118.7 (4)	C15—C14—H14A	109.4
C1—C2—H2	120.7	C13—C14—H14A	109.4
C3—C2—H2	120.7	C15—C14—H14B	109.4
C2—C3—C4	120.1 (4)	C13—C14—H14B	109.4
C2—C3—H3	119.9	H14A—C14—H14B	108.0
C4—C3—H3	119.9	O1—C15—C14	109.8 (5)
C3—C4—C5	118.4 (4)	O1—C15—H15A	109.7
C3—C4—H4	120.8	C14—C15—H15A	109.7
C5—C4—H4	120.8	O1—C15—H15B	109.7
N1—C5—C4	121.4 (4)	C14—C15—H15B	109.7
N1—C5—C6	115.4 (4)	H15A—C15—H15B	108.2
C4—C5—C6	123.2 (4)		
			
N3—Cu1—N1—C1	−171.0 (5)	O2—Cu1—O5—N5	−179.4 (3)
N2—Cu1—N1—C1	167.4 (4)	C5—N1—C1—C2	1.4 (6)
O2—Cu1—N1—C1	23.7 (4)	Cu1—N1—C1—C2	−177.4 (3)
O5—Cu1—N1—C1	−53.8 (4)	N1—C1—C2—C3	1.4 (7)
N3—Cu1—N1—C5	10.1 (8)	C1—C2—C3—C4	−2.4 (7)
N2—Cu1—N1—C5	−11.4 (3)	C2—C3—C4—C5	0.8 (7)
O2—Cu1—N1—C5	−155.2 (3)	C1—N1—C5—C4	−3.2 (6)
O5—Cu1—N1—C5	127.4 (3)	Cu1—N1—C5—C4	175.8 (3)
N1—Cu1—N2—C7	148.6 (3)	C1—N1—C5—C6	173.8 (4)
N3—Cu1—N2—C7	−26.0 (3)	Cu1—N1—C5—C6	−7.3 (5)
O2—Cu1—N2—C7	−115.3 (3)	C3—C4—C5—N1	2.1 (7)
O5—Cu1—N2—C7	59.1 (4)	C3—C4—C5—C6	−174.6 (4)
N1—Cu1—N2—C6	26.4 (3)	C7—N2—C6—C5	−152.9 (4)
N3—Cu1—N2—C6	−148.3 (3)	C13—N2—C6—C5	79.9 (4)
O2—Cu1—N2—C6	122.5 (3)	Cu1—N2—C6—C5	−36.0 (4)
O5—Cu1—N2—C6	−63.1 (3)	N1—C5—C6—N2	30.1 (5)
N1—Cu1—N2—C13	−92.1 (3)	C4—C5—C6—N2	−153.0 (4)
N3—Cu1—N2—C13	93.3 (3)	C6—N2—C7—C8	149.5 (4)
O2—Cu1—N2—C13	4.0 (4)	C13—N2—C7—C8	−83.5 (4)
O5—Cu1—N2—C13	178.4 (2)	Cu1—N2—C7—C8	33.1 (4)
N1—Cu1—N3—C12	169.3 (5)	C12—N3—C8—C9	1.5 (6)
N2—Cu1—N3—C12	−169.2 (4)	Cu1—N3—C8—C9	178.5 (3)
O2—Cu1—N3—C12	−25.3 (4)	C12—N3—C8—C7	−175.2 (4)
O5—Cu1—N3—C12	51.7 (3)	Cu1—N3—C8—C7	1.8 (5)
N1—Cu1—N3—C8	−7.5 (8)	N2—C7—C8—N3	−24.4 (6)
N2—Cu1—N3—C8	14.0 (3)	N2—C7—C8—C9	159.0 (4)
O2—Cu1—N3—C8	157.9 (3)	N3—C8—C9—C10	−2.8 (7)
O5—Cu1—N3—C8	−125.0 (3)	C7—C8—C9—C10	173.6 (4)
O4—N4—O2—Cu1	173.9 (3)	C8—C9—C10—C11	1.8 (7)
O3—N4—O2—Cu1	−4.6 (5)	C9—C10—C11—C12	0.4 (7)
N1—Cu1—O2—N4	79.6 (3)	C8—N3—C12—C11	0.8 (6)
N3—Cu1—O2—N4	−96.7 (3)	Cu1—N3—C12—C11	−175.8 (3)
N2—Cu1—O2—N4	−11.4 (4)	C10—C11—C12—N3	−1.8 (7)
O5—Cu1—O2—N4	172.4 (3)	C7—N2—C13—C14	−71.2 (5)
O7—N5—O5—Cu1	177.9 (4)	C6—N2—C13—C14	57.7 (5)
O6—N5—O5—Cu1	−2.2 (5)	Cu1—N2—C13—C14	172.7 (3)
N1—Cu1—O5—N5	−81.4 (3)	N2—C13—C14—C15	172.9 (4)
N3—Cu1—O5—N5	85.7 (3)	C13—C14—C15—O1	62.6 (6)
N2—Cu1—O5—N5	3.9 (4)		

### Hydrogen-bond geometry (Å, °)

**Table:**

*D*—H···*A*	*D*—H	H···*A*	*D*···*A*	*D*—H···*A*
O1—H1···O7^i^	0.84	2.18	2.961 (6)	155

Symmetry codes: (i) *x*+1/2, −*y*+1/2, *z*+1/2.
